# The Transdiagnostic Relevance of Self-Other Distinction to Psychiatry Spans Emotional, Cognitive and Motor Domains

**DOI:** 10.3389/fpsyt.2022.797952

**Published:** 2022-03-10

**Authors:** Clare M. Eddy

**Affiliations:** Birmingham and Solihull Mental Health NHS Foundation Trust, College of Medical and Dental Sciences, University of Birmingham, Birmingham, United Kingdom

**Keywords:** empathy, social cognition, self-other distinction, Tourette syndrome, obsessive-compulsive disorder, schizophrenia, autism, personality disorder

## Abstract

Self-other distinction refers to the ability to distinguish between our own and other people's physical and mental states (actions, perceptions, emotions etc.). Both the right temporo-parietal junction and brain areas associated with the human mirror neuron system are likely to critically influence self-other distinction, given their respective contributions to theory of mind and embodied empathy. The degree of appropriate self-other distinction will vary according to the exact social situation, and how helpful it is to feel into, or remain detached from, another person's mental state. Indeed, the emotional resonance that we can share with others affords the gift of empathy, but over-sharing may pose a downside, leading to a range of difficulties from personal distress to paranoia, and perhaps even motor tics and compulsions. The aim of this perspective paper is to consider how evidence from behavioral and neurophysiological studies supports a role for problems with self-other distinction in a range of psychiatric symptoms spanning the emotional, cognitive and motor domains. The various signs and symptoms associated with problematic self-other distinction comprise both maladaptive and adaptive (compensatory) responses to dysfunction within a common underlying neuropsychological mechanism, compelling the adoption of more holistic transdiagnostic therapeutic approaches within Psychiatry.

## Introduction

### What Is Self-Other Distinction and Why Is It Important?

Humans are innately wired to respond to others' emotional states. Most of us understand what it is to vicariously feel other's pain, and if we are lucky, their happiness. This emotional resonance that we can share with others appears automatic, and its greatest gift is that of affective empathy and our ability to respond sensitively to the needs of others. However, successful navigation of the social world also requires that we can contemplate the contrasting perspectives of self and other, and too much sharing may have a downside.

Self-other distinction refers to the ability to distinguish between our own and other people's physical and mental states, including actions, perceptions, emotions etc. Low self-other distinction (or self-other blending/merging) is associated with processes that contribute to the recognition of mental states in others including imitation or mirroring. However, low self-other distinction can be associated with misattributions of mental states when these differ for self and other, a situation when abstract mentalizing that holds in mind these opposing perspectives is important. Mirroring tends to occur in response to visual stimuli and for embodied mental states, whereas mentalizing is critical when visual cues to mental states are potentially misleading or not readily observable (e.g., verbal tasks; understanding beliefs; deception etc.). The appropriate degree of self-other distinction will therefore vary according to the exact social situation e.g., affective empathy may involve low self-other distinction, whereas understanding false belief requires higher self-other distinction (Table 1).

**Table 1:** Self-other distinction and two approaches to social cognition.

**Mirroring**	**Mentalizing**
Embodied: external focus of attention	Abstract: internal focus of attention
Relies on non-linguistic cues (expression/tone)	Can rely on linguistic cues
Innate, more automatic	Develops in childhood, more effortful
Concrete: mental states must be directly inferred from action	Theoretical: accommodates unobservable mental states
Motion, emotion	Cognition
Reflexive/reactive—lower self control	Calculating/reasoning—higher self control
Emotion contagion, affective empathy	Cognitive empathy, theory of mind
Observer resonates with and becomes one with the externally perceived other: sense of a separate self is momentarily lost (low self-other distinction)	Observer actively constructs imaginary representation of other within the larger construct of the self: maintaining separate self (higher self-other distinction)

### Which Brain Regions Contribute to Self-Other Distinction?

One neural network highly relevant to self-other distinction is the Mirror Neuron System [MNS: (1)] which was first identified in primates (2–4). The MNS is thought to underpin motor simulation of observed actions, providing a basis for imitation, and to draw upon visual cues to support the understanding of action goals [e.g., (5, 6)], in turn facilitating inferences about emotions, and perhaps beliefs and intentions (7, 8), although its exact contribution to empathy continues to be investigated (9). In humans, the MNS includes the inferior frontal gyrus (BA44/45), superior temporal and inferior parietal lobe (10–12). This network develops very early in childhood (13), and may be automatically activated before the second network, the mentalizing system (14, 15), which includes the medial prefrontal cortex, precuneus, and right temporal parietal junction (rTPJ). The mentalizing network supports more conscious reasoning about mental states (15, 16).

The rTPJ is of particular importance in self-other distinction, given its role in multi-sensory integration and sense of embodiment (17), and activation during tasks where the differing beliefs of self and other are salient (18, 19). Further evidence comes from the effects of rTPJ stimulation on tasks that require self-other distinction such as imitation inhibition tasks (20–22). Most studies suggest that rTPJ activity is positively associated with self-other distinction (8), such that activations may emphasize incongruence between self and other, or allow for switching between their related representations [e.g., (23)].

### What Might Happen If Self-Other Distinction Goes Wrong?

Within the social cognitive domain, indicators of low self-other distinction include motor imitation, and emotion contagion, when we effectively take on the physical and emotional states of others. These acts of mirroring encourage the automatic sacrifice of a sense of separate self as the observer becomes one with the perceived other. This loss of self-other distinction could be less likely to occur in the context of mentalizing, which may involve the conscious and controlled construction of an imaginary other (or alternative self) perhaps subordinate to and easily distinguishable from the one's true self. In sum, sense of oneself as a unique individual entity, and as the originator or controller of perceived internal and external states (e.g., actions and emotions), may be vulnerable to the effects associated with loss of self-other distinction and the mirroring experience. On the other hand, in some cases, too much self-other distinction could be problematic.

The aim of this perspective paper is to synthesize the evidence suggesting that problems with self-other distinction are relevant to the development of numerous psychiatric disorders, building on previous research (8, 17) through the integration of additional evidence in the form of both behavioral and neurophysiological studies within the field of psychiatry. While many factors may influence self-other distinction (e.g., executive dysfunction; self-efficacy; sensory impairments), this opinion piece focuses on processes that are typically associated with mirroring, with reference to the contrast with conscious mentalizing. Key questions were: 1. Is it possible to identify primary (direct) vs. secondary (indirect) signs of problematic self-other distinction? 2. Are there secondary signs with opposing/compensatory effects? I will argue that a range of clinical symptoms across emotional, cognitive and motor domains constitute various manifestations of impaired self-other distinction, resulting from dysfunction within a common underlying neural mechanism, with important implications including in terms of treatment approaches.

## Relevance of Self-Other Distinction to Psychiatry

### Self-Other Distinction Within the Emotional Domain

The primary cause of loss of self-other distinction within the emotional domain is likely to be high emotion contagion. MNS responses are emotion specific and more sensitive to negative valence (24, 25), therefore excessive resonance with others experiencing negative emotion is likely to result in increased personal distress. High personal distress is common in psychiatric disorders but is not usually accompanied by high empathic concern (Table 2). Perhaps continued experience of personal distress can prove aversive, leading individuals to self-report lower empathic concern as they become more focused on resolving their own internal emotional state. The unpleasantness of excessive emotional resonance could also contribute to social anxiety and social anhedonia (8). Furthermore, the relationship between performance on social cognitive tasks and emotional resonance may fall on an inverted U-curve helping to explain patterns of social cognitive performance (e.g. inconsistent impairment across tasks) in numerous psychiatric disorders (8, 17).

**Table 2:** Evidence for problems with self-other distinction in psychiatric disorders.

**Domain**	**Symptom/sign**	**Disorder**	**Study findings**
Emotional	Emotion contagion*	TS	Heightened neural response to facial expressions (26, 27)
		SZ	Higher than HCs (28). Empathizing v systematizing bias associated with paranoia (29)
		OCD	Higher emotional response to observed emotions (30)
		ASD	Can be lower than HC but influenced by target familiarity and eye gaze (31). Emotion contagion for pain is intact (32)
		BPD	Higher that HCs (33) with one study showing this using EMG while patients viewed negative facial expressions (34)
		NPD	Mix of no difference/lower self-report in association with grandiose subtype traits in non-clinical sample (35)
	Personal distress	TS	Higher personal distress (but lower IRI perspective taking) than HCs (36)
		SZ	Higher personal distress (but lower IRI perspective taking) than in controls (37). Personal distress positively related to symptoms (38)
		OCD	Higher personal distress than HCs (39) and lower perspective taking (40)
		ASD	Higher personal distress than HCs (41). Autistic traits linked to high personal distress in general population (42)
		BPD	Higher personal distress than HCs (43–47)
		NPD	High personal distress in covert/vulnerable narcissism (48, 49)
	General emotion dysregulation	TS	Correlates with tic severity (50), high in more complex cases (51)
		SZ	Overwhelming/lack of control over emotions (28); mediates symptom expression (52, 53)
		OCD	Heightened affective responses and poor emotion regulation, but perhaps lower motor resonance (54)
		ASD	High levels in autism (55–57) and Asperger's (58)
		BPD	Low cognitive empathy in high vs. low borderline traits, associated with emotional dysregulation (59)
		NPD	Rivalry (60) and vulnerable narcissism associated with more problems vs. grandiose (35, 61–63)
	Social anxiety/ social anhedonia	TS	Higher social anxiety than HC (64). Attentional bias toward social threat (65)
		SZ	Linked to perception of negative valence in facial expressions (66, 67) and empathy/emotion contagion (68)
		OCD	Higher social anxiety than HC (69). Linked to altered activity in right STG (70)
		ASD	Both seen in adults (71); social anxiety in adolescents (72); social anhedonia correlates with autism severity (73)
		BPD	High social anxiety in clinical sample (74) and associated with traits (75)
		NPD	More likely in vulnerable narcissism (76)
	Alexithymia^†^; flat/ constricted affect	TS	May be related to strength of sensory urges to tic (77, 78)
		SZ	Difficulty describing and identifying feelings (79). Flat affect related to ToM tasks (80), despite heightened automatic sensitivity to facial affect (81), increased amygdala reactivity (82) and altered IPL activity (83)
		OCD	Higher alexithymia than HCs (84, 85) and more blunted affect (69). Associated with mental neutralizing (86) and suicide risk (87)
		ASD	High alexithymia (88) associated with emotional dysregulation (56). Reduced facial expression in children (89, 90)
		BPD	Higher alexithymia than HCs (46, 91–94). Linked to non-suicidal self-injury (95). Less facial expression of emotion (96)
		NPD	Seen in clinical and non-clinical samples and associated with empathy (97–99)
Cognitive	Misattribution of origin of mental states i.e., projection; paranoia; hyper-mentalizing	TS	Projection could explain performance on ToM tasks (77, 100). Some paranoid thoughts more common than in HC (101, 102)
		SZ	Projection could explain performance on ToM tasks e.g., attributions of mental states to non-social stimuli [e.g., (103)] and neutral expressions appearing negative (104). Hyper-mentalizing errors (105) including self-referential hyper-mentalizing in schizotypy (106)
		OCD	Paranoia associated with OCD symptoms in non-clinical (107) and clinical (69, 108) samples. Hoarding associated with anthropomorphising (109)
		ASD	Autistic traits associated with anthropomorphising (110, 111). Characteristics linked to paranoia (112, 113) and persecutory ideation (58) can present
		BPD	Projection and projective identification (114). Paranoia (115) including more severe non-delusional paranoia than SZ (116)
		NPD	Paranoia associated with low mood (117), rejection sensitivity (118) and the proposed diagnosis of malignant narcissism (119, 120)
	Difficulty with self (i.e., coherent, stable self concept)	TS	Lower self-concept reported in TS (121) or TS+OCD (64) although measures seem closely related to self-esteem
		SZ	Poorer self-definition and negative self-regard (122) linked to emotional experience (123). Fundamental loss of sense of self (124)
		OCD	Sensitive self-concept, negative view of self (125, 126) or feared self (127)
		ASD	Weaker self-concept (128, 129) and hoarding has been suggested to help maintain continuity of self in autistic spectrum (130)
		BPD	Identity confusion (131, 132) and self and other representational disturbances (133, 134)
		NPD	Impaired sense of self (135) including lack of integration of self (136)
	Altered sense of agency/magical thinking	TS	Jumping to conclusions bias (137) and greater tendency than controls to ascribe intentions to randomly moving shapes (77). Symptoms of OCD (which often include magical thinking) frequently comorbid with TS (138)
		SZ	Tendency to thought action fusion (139). Alterations to self agency and relatedness (122) and decreased sense of self-causation (140). Lower sense of agency in high schizotypal non-clinical sample (141)
		OCD	Tendency to thought action fusion (142, 143). Belief that one has excessive ability and responsibility to prevent harm (144). Lower use of agency related language vs. HCs (145)
		ASD	Reduced intention attribution (146) and altered sense of agency in mystical experience (147)
		BPD	BPD v HC less agentic in their descriptions for self and other stories seeing people as powerless (148).
		NPD	High vs. low sense of agency and self-esteem associated with grandiose traits vs. vulnerable traits respectively, in non-clinical sample (149)
	Reduction in conscious perspective taking^†^	TS	Lower self-reported perspective taking vs. HCs (36)
		SZ	Lower self-reported perspective taking vs. HCs (37)
		OCD	Lower self-reported perspective taking vs. HCs (40)
		ASD	Problem with explicit perspective taking but not necessarily empathy (150)
		BPD	Cognitive perspective taking can be reduced (151)
		NPD	Most likely to be reduced when affect is involved and may depend on subtype (152–154)
	Antagonistic (including egodystonic) impulses and actions^†^	TS	Coprophenomena and non-obscene socially inappropriate urges that tend to be ego-dystonic (155–157)
		SZ	Impulsive non-conformity is associated with atypical emotional prosody processing (158); high in schizotypy in association with reasoning about actions based on emotions (159); negatively correlated with anhedonia (160)
		OCD	Ego-dystonic intrusive thoughts about harming others (161) associated with proposed ‘self-defeating' personality disorder (162, 163)
		ASD	Acute agitation and aggression (164) and problem behaviors which may be related to coping skills (165)
		BPD	Emotional dysregulation linked to splitting, projection and acting out (166). Low compliance (167) and self-defeating behavior (168)
		NPD	Antagonism is at the core of narcissism (169, 170) low compliance (167) and self-defeating traits (171) are also associated
	Narcissism/ grandiosity^†^	TS	Features linked to vulnerable narcissim more likely to occur and associated with depression (172)
		SZ	Grandiosity may have a defensive or protective role (173, 174)
		OCD	Can get a proportion of people with obsessive-compulsive traits who are diagnosed with NPD (175)
		ASD	NPD can be co-morbid (176) and tendency to self-enhance (177)
		BPD	Vulnerable traits are more closely related (178–180)
		NPD	Grandiosity is often central to NPD, though less prominent in vulnerable than grandiose subtype (149, 170, 181)
Motor	Echophenomena/excessive motor resonance*	TS	Echophenomena are characteristic of TS (182, 183). Severity associated with TPJ activity during social cognitive tasks (26, 184). Poor inhibition of imitation (185, 186)
		SZ	Echophenomena classified as a form of catatonia and seen in drug naïve cases (187, 188). Both enhanced (189) and impaired imitation (190): effort/medication may influence
		OCD	Reported deficits in imitation of meaningless movements (191) vs. contrasting evidence of more vicarious experience from others' movement (30). OCD is often comorbid with TS
		ASD	Echophenomena may present (192, 193). Greater automatic imitation associated with reduced activity in med PFC and TPJ in autism (194)
		BPD	Higher MEG response to facial expressions (34). Poor imitation inhibition i.e., interference from observed movements (195)
		NPD	Stronger motor-emotional resonance when observing physical pain despite lower self-reported empathy (196)
	Sensing loss of agency over movements/ actions	TS	Sense of tics as being involuntary [e.g., (197)], and reduced accuracy in action monitoring (198–200)
		SZ	Delusions of control over actions seen in association with psychosis (201). Greater susceptibility to illusions of body ownership in schizotypy (202)
		OCD	Low intentional binding but higher illusory control (203). Altered sense of motor agency (204, 205)
		ASD	Larger temporal window of integration and potential excessive binding between unrelated stimuli (206). In addition, reduced intentional binding may be seen (207), perhaps affecting sense of agency (208)
		BPD	Greater susceptibility to illusions of body ownership vs. HC (195, 209–211) but can self-report higher sense of agency (210)
		NPD	Narcissistic traits have a positive relationship with intentional binding and sense of agency (212) despite link to impulsivity (213), which may reflect grandiose vs. vulnerable difference (149)
	Motor compulsions (including tics)^†^	TS	Motor compulsions include symmetry and evening up compulsions (214), and self-injurious behavior [e.g., (215)], plus more general difficulties with motor inhibition [e.g., (216)]
		SZ	Tics can precede typical symptoms of SZ and related treatments [e.g., (188, 217)]
		OCD	Compulsions are related to sensori-motor issues (205, 218). Reduced motor inhibition/enhanced tendency to action (219)
		ASD	Tics (220) and motor stereotypies and compulsions are often present, including self-injurious behaviors (221, 222)
		BPD	Impaired motor inhibition related to general impulsivity and dissociation (223, 224). Self-harm linked to compulsivity (225)
		NPD	Occasionally associated with exercise (226) and sexual (227) compulsions but not simple motor compulsions
	Impulsivity	TS	Impulsive behaviors are common in TS (228) and can involve self-harm (215). There may be a predisposition toward motor impulsivity in general (229, 230)
		SZ	Impulsive behaviors can occur in response to command hallucinations (231). Less impulsive than BPD or OCD (232) but impulsive non-conformity linked to risk-taking behavior in schizotypy (233)
		OCD	Motor impulsivity linked to hoarding symptoms (234) but most behaviors more closely linked to compulsivity (235)
		ASD	Impulsivity linked to self-injurious behavior (236, 237)
		BPD	Phenotypic trait according to longitudinal studies (238). High impulsivity (239, 240) especially if in negative emotional state (241) related to alexithymia (242) and anhedonia (243)
		NPD	Linked to impulsive buying (244) but may vary according to subtype (149, 245, 246)
Neuro	MNS: Atypical activity/ structure	TS	Atypical activity within IPL/TPJ and IFG during observation of facial expressions (26), altered structural connectivity between these areas, basal ganglia and thalamus (247), and lower volume of IFG (248).
		SZ	Greater MNS activity when observing movement in association with psychosis (249), linked to both positive (250) and negative (251) symptoms. Resting state connectivity is also atypical (252, 253)
		OCD	Altered activity in MNS regions when perceiving biological motion (254). Structural changes in IPL (58, 255) and IFG (58, 256) and thickness of right IFG can be associated with symptoms (257)
		ASD	IPL responses negatively correlated with autism symptom severity in adults (258) and MNS abnormalities include reduced IFG activity (259, 260)
		BPD	Atypical activity in frontal and/or parietal components of the MNS (261–263) including during pain processing (264) and emotion contagion (265)
		NPD	EEG differences to HC during empathy for pain involving somatosensory cortex (196). Reduced cortical thickness in right IFG (266)
	rTPJ: Atypical activity/ structure	TS	Hyperactive for facial expressions (26) but hypoactive during false belief task (184). Activity correlates with echophenomena and global tic severity (26, 184). Atypical structural connectivity (247, 267). Atypical activity for imagined and executed movements (268)
		SZ	Hyperactive during ToM task when high risk (269); hypoactive after diagnosis (269, 270). Hypoactive during other vs. self reflection (271) and during naturalistic social cognitive tasks (272). Functional connectivity and structural differences to HC (273, 274)
		OCD	Altered resting state functional connectivity (275) including MEG study (276). Increased volume (58)
		ASD	Dysfunction during imitation (277), observation of social interaction (278, 279), belief reasoning (280) and perspective taking (281). Reduced selectivity for mental vs. physical states (282). Activity linked to impaired communication (283)
		BPD	Both hypoactivity during perspective taking (284) and hyperactivation while evaluating own and others' personality traits (285)
		NPD	No studies identified (few imaging studies overall)

Proposed to result from low self-other distinction*; may help to increase self-other distinction^†^.

*ASD, autistic spectrum disorder; BPD, borderline personality disorder; EEG, elctroencephalography; EMG, electromyography; HC, healthy controls; IFG, inferior frontal gyrus; IPL, inferior parietal lobe; IRI, Interpersonal Reactivity Index; MEG, magnetoencephalography; MNS, mirror neuron system; NPD, narcissistic personality disorder; OCD, obsessive-compulsive disorder; PFC, prefrontal cortex; SZ, schizophrenia; ToM, theory of mind; rTPJ, right temporo-parietal junction; TS, Tourette syndrome*.

Frequent unregulated emotional contagion may encourage confusion around the source (self/other) of experienced emotional states. Alexithymia (286), or difficulty identifying and expressing emotions, could be one consequence of this confusion stemming from vicarious experience of other's emotions in the absence of a linking situational cause for that emotion in oneself (8). However, alexithymia could indicate reduced attention to internal states which in turn reduces the salience of excessive emotional resonance or personal distress (8, 287). Other forms of emotional blunting (e.g., constricted/flat affect), and perhaps dissociation, could support similar regulatory functions in terms of avoiding exposure to problematic emotions of self and/or other. Such emotional responses may be largely unconscious conditioned responses to the primary problem of loss of self-other distinction within the emotional domain.

Some psychiatric disorders feature anti-social behaviors which should prompt an emotional reaction in others, such as the compulsive socially inappropriate urges seen in Tourette syndrome (TS). TS is associated with heightened personal distress and increased emotional reactivity to emotional facial expressions (26, 36), and patients who experience urges to make offensive remarks/gestures find them troubling as they don't consciously wish to cause distress (288). On the surface, socially inappropriate actions imply emotional disregard, and emphasize self-other distinction because the patient's transgression is in direct antagonism to the others' emotional needs (8) i.e., the anti-social action (at least momentarily) separates the perpetrator from the victim because the intention and action goals associated with their anti-social act conflict with the desired mental state of the victim. However, in addition to counteracting any feeling of excessive emotional resonance, such actions promote control over the emotional state of others. Therefore, rather than emphasizing self-other distinction, anti-social urges could arise from an unconscious urge to prompt a negative emotional mental state within another that matches the patient's own negative internal state (i.e., reduced self-other distinction). This may provide a better explanation for some emotionally provocative and antagonistic behaviors seen in Borderline Personality Disorder (BPD) and Narcissistic Personality Disorder (NPD).

### Self-Other Distinction Within the Cognitive Domain

Excessive emotional resonance with others and arising difficulties with self-other distinction could have a broader effect on conscious experience of cognitive mental states including judgments about the origin of these. Difficulty knowing whether a thought or intention arose from the self explains many symptoms of psychosis [e.g., (155)] including delusions relating to thought transfer and telepathy. Incorrect assumptions that one is aware of the cognitive mental state of another could also reduce mentalizing leading to egocentric errors (289). Projection of negative emotions or intentions onto others, as seen in disorders such as BPD and schizophrenia (including on social cognitive tasks: Table 2), is likely to prompt social anxiety and paranoia. If a projected thought is positive, it could encourage grandiosity. Doubts about whether thoughts are internally generated may also underlie magical thinking as seen in Obsessive-compulsive Disorder (OCD), explaining the association between negative sense of agency and likelihood thought action fusion (287) i.e., the belief that thinking about events makes those events more likely to happen.

In some cases, loss of self-other distinction may weaken the stability of our overall conscious construct of self, as most clearly seen in BPD and schizophrenia. When this occurs, it appears all the more important to develop cognitive strategies that help restore self-other boundaries. Strategies are likely to include conscious avoidance of mentalizing, helping to explain the low self-reported perspective taking that often accompanies high personal distress (Table 2), and perhaps poor performance on social cognitive tasks. In addition, impulsive non-conformity, whereby individuals with schizophrenia express strong opposition to convention and the opinions or expectations of others, even where this would seem harmful or irrational, may enhance cognitive self-other distinction. Similar characteristics can be seen in NPD, where rivalry and entitlement emphasize one's own uniqueness, and deception may be used to maintain differentiation between the cognitive mental states of self and other (152).

### Self-Other Distinction Within the Motor Domain

Excessive motor resonance in the form of echophenomena is likely to indicate loss of self-other distinction within the motor domain. Similar more subtle characteristics may be observed during imitation inhibition tasks, through magnetoencephalography, or perhaps when exploring susceptibility to the rubber-hand illusion (Table 2). Given the role of the MNS in emotion contagion there is likely to be a link between motor resonance and neural limbic response [e.g., (290, 291)], and therefore greater motor resonance and a tendency to emotional dysregulation (although MNS activity may not always manifest as observable movement). Difficulties in deciding whether the self is the agent of movements and related sensory events could help to explain the perception of involuntary movements, and perhaps depersonalization, in some psychiatric disorders. Weakened sense of ownership of personal actions could encourage impulsivity, and in more severe cases, delusions of control.

One proposed mechanism thought to influence self-other distinction is based on movement efference and predictive sensory feedback [e.g., (292, 293)], whereby dysfunction impairs determination of self-produced actions and effects, with relevance to conditions such as psychosis (294–296). Disrupted sensory feedback (alike excessive motor resonance) could have a conscious cognitive correlate in the form of altered sense of agency. Indeed, sense of agency appears to consist of both intrinsic (i.e., a more conscious, cognitive experience of agency) and extrinsic (i.e., sensorimotor experience of body ownership) aspects, and differences in integrating or balancing intrinsic and extrinsic self-representation networks could impair self-other distinctions (297).

Tics and compulsions can be associated with sensorimotor abnormalities (298, 299) and alterations in sense of agency for action (Table 2). While tics are reported as feeling at the most semi-voluntary, and tend not to appear goal directed, one effect of these internally generated fragments of motor activity is to interrupt motor resonance with external others, helping to support self-other differentiation, and perhaps developing into a habit conditioned to the experience of internal emotional stress. That is, the sensory fulfillment associated with tics and motor compulsions may arise through the acting out of a self-initiated action which helps to confirm (perhaps subconsciously) internal control over movement and related neural motor activity, counterintuitively helping to re-establish sense of agency. Given that both emotion and sense of self are relevant to self-harm (300), compulsive self-harm may be another symptom through which a self-initiated motor act enables a sense of self-control or internal agency over a perceived emotional or sensory state.

### Self-Other Distinction Within the Brain

Excessive resonance with others is perhaps most likely to be reflected in atypical activity within the MNS, as seen in disorders including TS and schizophrenia (26, 249). More generally, inferior parietal and inferior frontal activations have been shown to be atypical during social cognitive tasks in TS, ASD and BPD; unusual resting state activity has been revealed in schizophrenia; and structural changes have been associated with symptoms of OCD and NPD (Table 2). Problems with self-other distinction may also manifest as atypical activity within the mentalizing system, perhaps as hypo-activation of rTPJ when mentalizing is cued or hyper-activation when it is not [e.g., 29, 46]. Many studies have revealed that the right TPJ in particular, may demonstrate atypical activity during social cognitive tasks in patient populations with symptoms linked to problems with self-other distinction.

Perhaps the best evidence links brain dysfunction directly to behavioral signs of self-other distinction problems or related symptoms. For example, in TS, global measures of echophenomena and urges to tic have been associated with rTPJ activity during two different social cognitive tasks (26, 184). In schizophrenia, psychosis has been linked to negative symptoms (249) and excessive activity within the MNS (83), while reduced neural synchrony involving rTPJ has been implicated in impaired social communication in autism (283). Overall however, few studies have attempted to explore specific associations.

## Discussion

### Primary Effects, Secondary Symptoms and Coping Strategies

Many neuropsychiatric disorders feature emotional, cognitive and/or motor features that are likely to indicate problems with self-other distinction. Within each of these domains, we may identify both signs of low self-other distinction, and characteristics or behaviors that could constitute secondary effects or coping strategies which serve to increase self-other distinction. For example, frequent emotion contagion may lead to emotional dysregulation, and detachment from emotional experiences may combat personal distress. Cognitive features associated with poor self-other distinction may manifest as paranoia or projection, and potential coping strategies include avoidance of perspective taking or buffering sense of self through grandiosity or impulsive non-conformity. Excessive motor resonance with others (e.g., poor imitation inhibition) may reduce sense of physical agency and encourage the development of tics and compulsions that may help to restore this.

A novel contribution of the hypotheses presented herein is that they can account for a range of seemingly contradictory behaviors and self-defeating symptoms. There is irony in that many of the symptoms that arise through difficulties with self-other distinction, and reflect greater resonance with others' mental states, could appear to suggest hypo-mentalizing or antagonism toward others. This highlights the importance of considering both ability and application. Where over-application occurs, resulting difficulties may be as great as in cases of under-application.

While the concept of self-other distinction can be applied to cognition, emotion or movement, it's also important to consider automaticity, or implicit vs. explicit processes and skills, where possible. For example, processes that reduce self-other distinction and involve the motor and limbic system (e.g., emotion contagion) appear fairly implicit or automatic (301, 302), although some individuals may be more susceptible to the cues that initiate this. In contrast, complex higher level mentalizing may be to some extent more explicit or controllable (16, 186, 303). An over-responsive MNS leading to frequent limbic dysregulation may initiate confusion around sense of agency, which then becomes more generalized to thought and action. In general, as we cannot directly observe another person's thought, it makes sense for cognitive signs to occur further downstream. For example, while excessive automatic emotion contagion is often a primary sign, secondary effects such as reduced perspective taking or conscious attention to other's emotions, may help to compensate for the primary problem (i.e., low self-other distinction). Other indirect signs (e.g., tics and motor compulsions) may seem less conscious, although differentiating between conscious strategies and automatic compulsive responses can be challenging. Furthermore, regulatory or compensatory effects may occur across domains, supported by the finding that both cognitive (thought action fusion; sense of agency) and emotional (personal distress) factors mediate the relationship between emotion contagion and alexithymia (287).

### Therapeutic Implications, Limitations and Remaining Questions

The struggle to achieve a healthy and functional balance of self-other distinction may manifest in a range of forms, from tics in TS, to repetitive cycles of affiliation followed by antagonism in BPD. The theory presented suggests while those with neurodevelopmental, anxiety and personality disorders express differing constellations of internalizing and externalizing symptoms, overlapping difficulties with self-other distinction imply shared dysfunction within a common underlying neuropsychological mechanism. Therefore the potential therapeutic benefit of addressing difficulties with self-other distinction should be extensive, once the specific associations between self-other distinction and the suggested related symptoms and coping mechanisms have been established. Psychological interventions have begun to consider factors which overlap with the self-other distinction theme (e.g., self-awareness; emotion regulation; mentalizing), including metacognitive approaches for psychosis [e.g., (304, 305)], and personality disorders (306, 307). Other emerging interventions combine non-invasive brain stimulation with social cognitive (308) or sensori-motor (309) related training. Future related research should seek to first fully define and operationalise the construct of self-other distinction, before identifying reliable measures (e.g., self-other overlap index) that can be used in assessment and evaluation. Ultimately we should seek to harness what we can from behaviors that appear to counteract a problem with self-other distinction in order to inform therapeutic strategies.

The proposed hypotheses prompt further unanswered questions. For example, longitudinal studies are necessary to test whether suggested primary signs of low self-other distinction (e.g., emotion contagion; echophenomena) precede the development of other symptoms such as alexithymia, blunted affect, paranoia, antagonistic behaviors. This would identify risk factors and targets for early intervention. While there should be common overlap in the underlying mechanisms, individual differences in neural organization or stage of development of self-other distinction difficulties or compensatory responses, would help to explain the predominance of features within a given domain e.g., motor in TS vs. cognitive in schizophrenia. Diagnostic and therapeutic approaches would also be informed by a better understanding of the specific neural networks and structures involved, as well as factors such as the relationship between self-other distinction and executive dysfunction (e.g., cognitive flexibility). Can most of the symptoms described be linked to dysfunction of rTPJ, and is this synonymous with over-activation of the MNS or altered functional connectivity between the mirroring and mentalizing networks? Recent studies have revealed rTPJ activation in relation to forward predictions in both highly social (310) and less social (311) contexts, so further related clinical research using carefully selected experimental tasks is needed.

Many psychiatric symptoms appear likely to stem from low self-other distinction. However, some behavioral problems may reflect excessive self-other distinction as a primary effect. For example, the data on autism seems to suggest a mixed pattern, which could be linked to motor and/or MNS dysfunction [(312–314); but see (315)]. Social cognition is frequently impaired in movement disorder (316) and an impaired motor system will likely impair self-other distinction through loss of feedback between motor resonance and emotional processes (317). In relation to primary and secondary effects, primary psychopathy is thought to involve a fundamental deficit in affective empathy and therefore high self-other distinction, whereas secondary psychopathy may involve indirect symptoms or those arising through a coping strategy (318). It is possible that some of the signs and symptoms presented here that are suggestive of high self-other distinction constitute primary rather than secondary effects. Furthermore, some behaviors could reflect either high or low self-other distinction [e.g., hypo-imitation: (319)] and whether an individual may fluctuate between polarized high or low self-other distinction (e.g., due to rTPJ dysfunction) remains to be explored. Other more general limitations include the challenges in reviewing the literature and drawing comparisons across different studies and disorders, because of variations in terms used, co-morbidities, reliability of self-report and unknown impact of medications.

## Conclusion

In conclusion, impaired self-other distinction, potentially underpinned by excessive mirroring, and/or hypoactivation of rTPJ, appears to lead to a disturbed sense of agency and the manifestation of a range of psychiatric symptoms across emotional, motor and cognitive domains. These symptoms variously reflect, or attempt to redress, the problematic level of self-other distinction. Understanding the hidden relationship between self-other distinction and symptoms as diverse as paranoia, self-harm, tics and narcissism, and considering the potential compensatory value of compulsive and antagonistic behaviors that are typically viewed as dysfunctional, will enhance our global understanding of mental health and expedite the development of more effective and innovative interventions.

## Data Availability Statement

The original contributions presented in the study are included in the article/supplementary material, further inquiries can be directed to the corresponding author/s.

## Author Contributions

The author confirms being the sole contributor of this work and has approved it for publication.

## Conflict of Interest

The author declares that the research was conducted in the absence of any commercial or financial relationships that could be construed as a potential conflict of interest.

## Publisher's Note

All claims expressed in this article are solely those of the authors and do not necessarily represent those of their affiliated organizations, or those of the publisher, the editors and the reviewers. Any product that may be evaluated in this article, or claim that may be made by its manufacturer, is not guaranteed or endorsed by the publisher.
